# Low-Voltage Electrical Burn of the First Web Space of the Hand

**Published:** 2013-03-01

**Authors:** Alexandra Condé-Green, Leigh Ann Price, Stephen M. Milner

**Affiliations:** Department of Plastic and Reconstructive Surgery, Johns Hopkins University School of Medicine, Balitmore, MD

## DESCRIPTION

**Figure 1:**
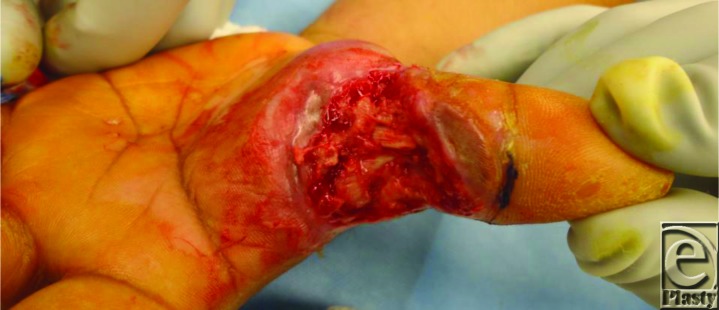
Full thickness electrical burn to the first web space of the left hand showing exposed tendons.

A 34-year-old right-hand-dominant man sustained an electrical injury to his left hand while handling a 450-volt cable. He presented with deep burns to the first web space and the dorsal aspect of the index finger. The excision of the eschar resulted in exposure of bare tendons (Fig 1).

## QUESTIONS

1. **What is the initial management of an electrical burn?**
2. **What is the management of the hand injury?**
3. **What are the reconstructive options?**
4. **A first dorsal metacarpal artery flap was performed on this patient. What are the common advantages and pitfalls?**

## DISCUSSION

Electrical injury often causes deep burns involving underlying soft tissue and bone. A careful history should be taken noting voltage and current involved, duration of contact with source, and related events (eg, loss of conscious, falls). Other associated complications include vascular and neurologic injuries, fracture, subluxation of joints, rhabdomyolysis and myoglobinuria, renal failure and cardiac arrhythmias.^1^ Primary and secondary surveys are completed, life-threatening injuries are eliminated, and the hand is temporarily dressed with saline gauze pending further assessment.

The general appearance of the hand is noted. Any deficit in range of motion, sensation, and regular neurovascular assessment is carried out. Whether or not immediate and definitive reconstruction should be attempted is clearly dependent on the circumstances of the accident, health of the patient, and consideration taken of the zone of injury.

Rapid resurfacing of the wound is essential to enable early mobilization and optimum recovery of hand function. Early reconstruction is favored when excision of all necrotic tissue is completed and allows for good wound coverage.^2^ Too early surgical intervention can lead to insufficient excision due to microvascular injuries and burn wound conversion.^3^ It can also lead to loss of potentially salvageable tissue, which may be pivotal at a later date for reconstruction, as reconstruction of any burn starts with the first operation. The optimal treatment is selected on the basis of the amount of tissue involved, the exposure of vital structures, the age, and relevant comorbidities of the patient. If the resulting tissue is limited to superficial soft tissue loss, a skin graft may be sufficient for coverage. Allograft may also serve as a temporary measure to allow better demarcation of the wound. However, if there is exposure of the deeper structures including loss of peritenon, the resulting defect should be closed with local or regional flaps or free tissue transfer.^4^ This young adult's injury is situated directly in the first web space. Because of its major contribution to overall hand function, correction of deformities involving the thumb is crucial.^5^^,^^6^

**Figure 2:**
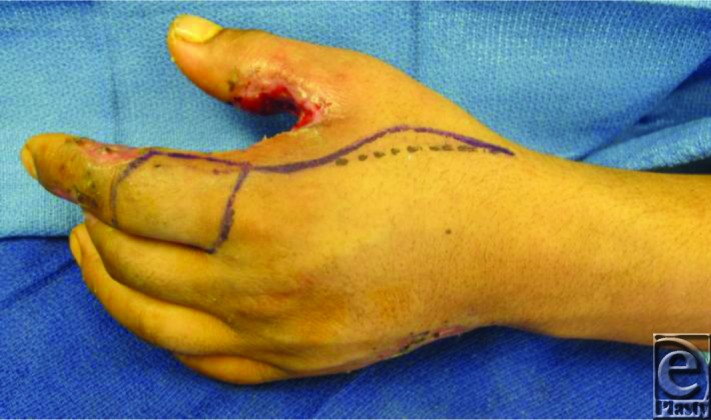
Marking of the course of the first dorsal metacarpal artery and flap.

The first dorsal metacarpal artery (FDMA) flap has become popular for this injury. This is an axial pattern skin flap extending proximally from the level of the metacarpophalangeal joint and distally to the level of proximal interphalangeal joint. The course of the artery is determined by Doppler and then marked. The flap is outlined on the proximal phalanx of the index finger extending between the mid lateral lines. A line is then drawn along the radial border of the second metacarpal in a lazy-S fashion, beginning from the lateral margin of the proximal base of the skin island to the tip of the first webspace. This represents the pivot point and the arc of rotation of the flap (Fig 2). The use of tourniquet makes this operative procedure essentially bloodless.

**Figure 3:**
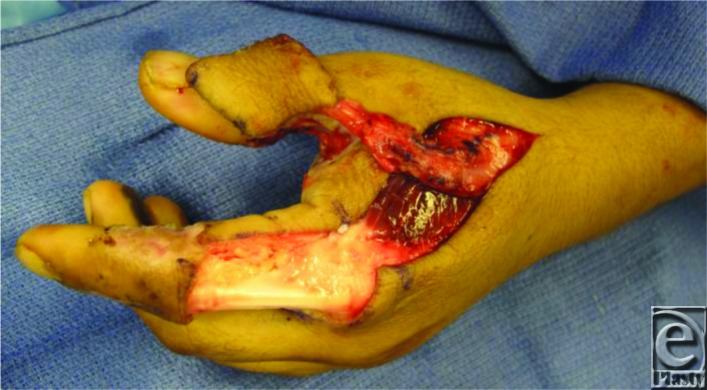
Pedicle and skin island of the FDMA flap.

**Figure 4:**
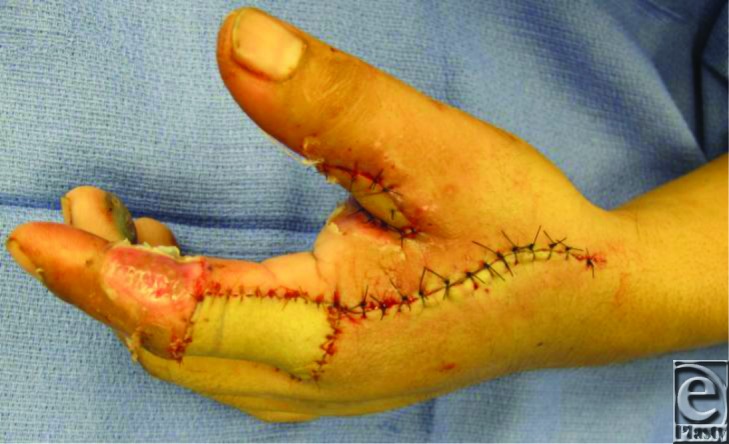
Immediate postoperative view of the FDMA flap.

**Figure 5:**
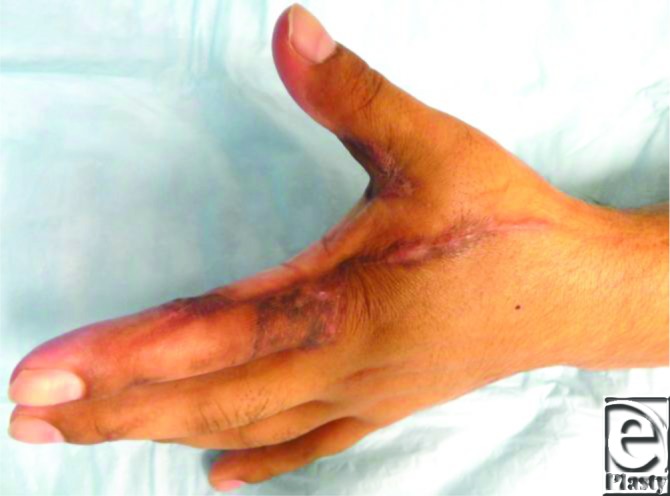
Six-month postoperative view of the FDMA flap, with thumb in full abduction.

Skin flaps are raised on both sides of the incision. Superficial veins that have the same course as the pedicle and the terminal branch of the dorsal sensory branch are incorporated with a cuff of superficial subcutaneous tissue. The fascia is then incised on the radial half of the first dorsal interosseous muscle proceeding toward the ulnar side in which the FDMA comes into view. Dissection proceeds to the periosteum at the dorsal radial edge of the second metacarpal including soft tissue around the FDMA to protect the pedicle. The skin island is elevated just above the extensor hood on the proximal phalanx of the index finger leaving the paratenon intact (Fig 3). The flap may be tunneled as in this particular case and sutured in place (Fig 4). The edges of the wound are approximated, and resurfacing of the defect on the dorsal aspect of the index finger is completed. The first dorsal metacarpal artery flap is a reliable, local, neurovascular island flap, offering good functional and esthetic outcomes (Fig 5). Furthermore, it has several advantages: an inconspicuous donor site, preservation of sensation, durability, and a wide rotation arch^7^ allowing early mobilization.
